# Perceived Benefits and Challenges of Coordinated Approaches to Chronic Disease Prevention in State Health Departments

**DOI:** 10.5888/pcd11.130350

**Published:** 2014-05-08

**Authors:** Peg Allen, Sonia Sequeira, Leslie Best, Ellen Jones, Elizabeth A. Baker, Ross C. Brownson

**Affiliations:** Author Affiliations: Sonia Sequeira, Washington University in St. Louis, St. Louis, Missouri; Leslie Best, Pennsylvania Department of Health, Harrisburg, Pennsylvania; Ellen Jones, University of Mississippi Medical Center, Jackson, Mississippi; Elizabeth A. Baker, Saint Louis University, St. Louis, Missouri; Ross C. Brownson, Washington University in St. Louis and Washington University School of Medicine, St. Louis, Missouri.

## Abstract

**Introduction:**

Chronic disease prevention efforts have historically been funded categorically according to disease or risk factor. Federal agencies are now progressively starting to fund combined programs to address common risk. The purpose of this study was to inform transitions to coordinated chronic disease prevention by learning views on perceived benefits and challenges of a coordinated approach to funding.

**Methods:**

A national survey on evidence-based public health was conducted from March through May 2013 among state health department employees working in chronic disease prevention (N = 865). Participants were asked to rank the top 3 benefits and top 3 challenges in coordinating chronic disease approaches from provided lists and could provide additional responses. Descriptive analyses, χ^2^ tests, and analysis of variance were conducted.

**Results:**

The most common perceived benefits of coordinated approaches to chronic disease prevention were improved health outcomes, common risk factors better addressed, and reduced duplication of program efforts. The most common perceived challenges were funding restrictions, such as disease-specific performance measures; competing priorities; lack of communication across programs; funding might be reduced; agency not structured for program coordination; and loss of disease-specific partner support. Rankings of benefits and challenges were similar across states and participant roles; the perceived challenges “lack of communication across programs” (*P* = .02) and “funding might be reduced” differed by program area (*P* < .001).

**Conclusion:**

Findings can be used by funding agencies and state health departments for planning, training, and technical assistance. The information on perceived challenges demonstrates the need to improve communication across programs, enhance organizational support for coordinated approaches, and create benefits for organizational partners.

## Introduction

The growing prevalence of chronic diseases with common risk factors has inspired a movement of coordinating chronic disease prevention practices (1–5). The rationale for coordinated approaches stems from the presence of multiple chronic conditions in many adults, the need for policies and environments to support health-enhancing behaviors to address common risk factors, and the need to reduce administrative burdens and duplicative efforts, as identified by state health departments and the Centers for Disease Control and Prevention (CDC) (4,6–10). Guiding principles for program coordination were jointly developed in 2006 by chronic disease prevention directors, CDC, and the National Association of Chronic Disease Directors (NACDD) (10).

Coordinated practice in chronic disease prevention by state health departments is intended to leverage resources to address common risk factors through evidence-based policies, programs, and services (5,8,10–13). Tobacco use, physical inactivity, poor nutrition, obesity, and low socioeconomic status are common risk factors for cardiovascular diseases, diabetes, and some types of cancer and are, therefore, ideal for coordinated approaches (Table 1) (4). Coordinated approaches may involve collaborating across program areas that historically have been funded categorically; reorganizing staffing patterns to apply core public health disciplines across multiple programs; collaborating with health care systems on system-wide policies; developing consistent messaging among organizations funded by the state health departments; and approaching problem solving collectively.

**Table 1:** Interrelationships Among Various Chronic Diseases and Modifiable Risk Factors, United States^a^^,^ b

Modifiable Risk Factor	Cardiovascular Disease	Cancer	Chronic Lung Disease	Diabetes	Cirrhosis	Musculoskeletal Diseases	Neurologic Disorders
Tobacco use	+	+	+			+	+
Alcohol use	+	+			+	+	+
High cholesterol	+						
High blood pressure	+						+
Poor diet	+	+		+		+	?
Physical inactivity	+	+		+		+	+
Obesity	+	+		+		+	+
Stress	+	?					
Environmental tobacco smoke	+	+	+				?
Occupation	+	+	+		?	+	?
Pollution	+	+	+				+
Low socioeconomic status	+	+	+	+	+	+	

a Source: Remington RL, et al, editors (4).

b Plus signs indicate that a positive relationship exists (eg, high blood pressure is associated with higher likelihood of developing cardiovascular disease); a question mark indicates that results are inconclusive to establish a relationship; and a blank cell indicates that no relationship exists.

Recent funding patterns for chronic disease prevention show a shift toward coordination. In 2009, CDC funded 53 state, tribal, and territorial health departments to collaborate for planning, infrastructure, interventions, and surveillance in diabetes, tobacco, and healthy communities (14). In 2009, CDC selected 4 pilot states to plan for coordinated chronic disease prevention (Colorado, Massachusetts, North Carolina, and Wisconsin) (15). From 2011 through 2014, all state health departments were funded to build capacity in coordinated chronic disease prevention (16). The CDC Coordinated State Support Branch provides technical assistance and consultation to states on coordination for 4 priority domains: epidemiology and surveillance, environmental approaches, health systems interventions, and community–clinical links. In 2013, CDC established combined funding for cardiovascular health, diabetes, obesity prevention, and school health programs (17). National data on coordinated practice for chronic disease prevention are limited.

The purpose of this article is to report perceived benefits and challenges to coordinated chronic disease prevention among a national sample of state health department staff working in chronic disease prevention.

## Methods

### Participants

State health department chronic disease prevention practitioners from the 50 US states and the District of Columbia completed an online survey from March through May 2013. Survey participants, identified from state health department websites or lists from partnering organizations, included program managers and staff in comprehensive cancer prevention and control, cancer screening, tobacco control, physical activity, nutrition, obesity prevention, diabetes prevention, and cardiovascular health. Human subjects approval was obtained from the institutional review board of Washington University in St. Louis.

### Measures

The survey was developed from the study team’s previous research, a literature review, and 5 rounds of study advisory group team input; survey development and testing are described elsewhere (18). In addition to 2 questions on coordinated chronic disease prevention, the 68-item survey contained items on respondent characteristics, evidence-based interventions, views on evidence-based decision making, training modality preferences and incentives, skill gaps, perceived agency support, and evidence resource access. For cognitive response testing, which improves survey development (19), we conducted 1-hour telephone interviews with 11 former employees of state health department chronic disease prevention programs. Participants discussed the meaning and clarity of each question. Reliability test–retest with 75 state health department staff from multiple states found adequate internal consistency (Cronbach’s α ≥ .70).

**Figure 1:**
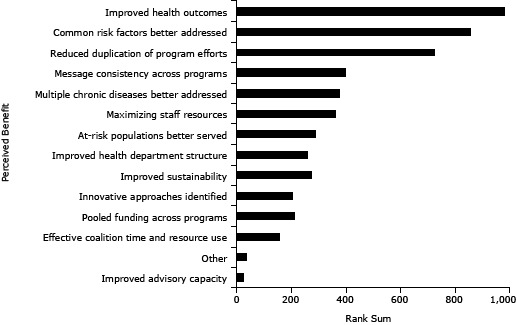
Perceived benefits of coordinated chronic disease approaches, United States, 2013. Participants (N = 865) in a national survey of state health department employees from all 50 states and the District of Columbia working in chronic disease prevention selected and ranked their top 3 anticipated benefits of coordinated chronic disease practice from the list shown in the figure. **Table:** 

**Figure 2:**
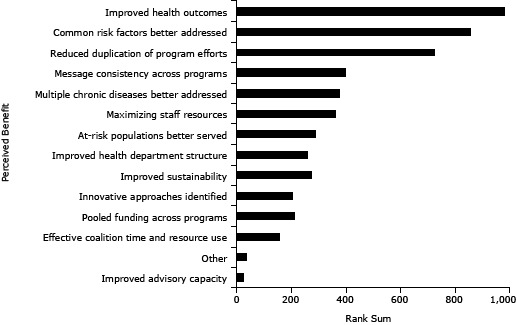
Perceived challenges of coordinated chronic disease approaches, United States, 2013. Participants (N = 865) in a national survey of state health department employees from all 50 states and the District of Columbia working in chronic disease prevention selected and ranked their top 3 challenges to coordinated chronic disease practice from the list shown in the figure. **Table:** 

Respondents were asked to rank their top 3 perceived benefits and challenges to coordinated chronic disease approaches from a list of response options. The questions were “What would you predict would be the most important potential benefits of coordinating/integrating chronic disease prevention programs?” and “What are the challenges to coordinating/integrating chronic disease prevention programs in your agency?” Instructions for both items were “Using the list below, please rank the top 3, where 1 is the most important” (Figures 1 and 2). Response options were drafted from public health practitioner discussions in previous evidence-based public health trainings (20). We revised the response options after feedback from 10 Missouri health agency coordinated chronic disease committee members and cognitive response testing. Reliability test–retest showed that 53.2% and 71.9% ranked at least 2 of the same 3 benefits and challenges response options, respectively, when taking the survey 2 to 4 weeks later. Participants could type in descriptions and rank “other” responses, which were grouped by 2 coders.

Perceived Benefit	Rank Sum
Improved health outcomes	980
Common risk factors better addressed	857
Reduced duplication of program efforts	726
Message consistency across programs	399
Multiple chronic diseases better addressed	380
Maximizing staff resources	362
At-risk populations better served	290
Improved health department structure	260
Improved sustainability	273
Innovative approaches identified	207
Pooled funding across programs	212
Effective coalition time and resource use	157
Other	34
Improved advisory capacity	25

Perceived Challenge	Rank Sum
Funding restrictions	1,054
Competing priorities	978
Lack of communication across programs	638
Funding might be reduced	563
Agency not structured for coordination	455
Disease-specific partners may not support	340
Staff resistance	267
Staff turnover	188
Loss of personal staff commitment	163
Lack of focus	156
Program impact decline	142
Other	107
Reduced public understanding	62

### Data analyses

Analyses were conducted using SAS version 9.3 (SAS Institute, Inc, Cary, North Carolina). Two descriptive statistics were calculated for each response option listed for perceived benefits and for perceived challenges. One statistic was the percentage of participants that chose the response option as 1 of their top 3. To calculate the second statistic, a rank sum, we assigned the item ranked first a score of 3, the second-ranked item a 2, and the third-ranked item a 1 for each individual, and then summed the scores for the item across all participants. We conducted χ^2^ or analysis of variance to compare participant characteristics across position (manager or director vs staff), program areas, and early coordinated chronic disease prevention planning states versus other states. Logistic regression was conducted with each of the most commonly ranked items as dependent variables in separate models adjusted for participant characteristics, chronic disease prevention funding levels in state health departments, chronic disease burden, and other state and health department characteristics. Archival data on characteristics of states and state health departments were obtained from the US Census Bureau, CDC, and the Association of State and Territorial Health Officials. Chronic disease funding was the total dollars CDC awarded to state health departments in fiscal year 2012. State chronic disease burden was calculated as the sum of *z*-scores (the number of standard deviations [SDs] above or below the mean values for each state) from mortality from the major cancer sites, cardiovascular disease, stroke, and chronic lung diseases; and prevalence of obesity, tobacco use, physical inactivity, and inadequate fruit and vegetable intake. Significance was set at *P* < .05.

## Results

### Participant characteristics

Of 1,169 state health department chronic disease practitioners invited from the 50 states and District of Columbia, 904 (77.3%) completed the survey. Complete information for the questions pertaining to the portion of the study described in this article was received for 865 participants. Of the 865, 80.5% were female, 70.2% had a master’s or doctoral degree, and 64.1% were program managers or directors. The mean number of years in public health was 14.8 (SD = 9.3 y) with a median of 13 years and a range of 1 to 46 years. Participants reported being in the state health department an average of 9.5 years (SD = 7.9 y) and in their current position for an average of 4.9 years (SD = 4.8 y).

### Perceived benefits of coordinated chronic disease prevention

Improved health outcomes, common risk factors better addressed, and reduced duplication of program efforts were the most commonly chosen and highest-ranked perceived benefits (Figure 1). Although participants could provide lengthy “other” text responses if desired, most typed brief phrases. “Other” text responses (n = 15) included

- “Increased focus on primary prevention”;
- “Increased support for common risk factor reduction”;
- “Unified messaging to the public and local partners”;
- “Synergies, including communities, partnerships”;
- “Programs can offer joint contracts with community-based organizations to implement services (vs 1 contract per program)”; and
- “Improved skills for EBDM” (evidence-based decision making).

The rankings of 2 commonly perceived benefits differed by program area (data not shown). Fewer participants in tobacco control (33.0%) or cancer prevention and control (34.4%) chose “common risk factors better addressed” in their top 3 items than participants from diabetes or cardiovascular health (50.0%) or obesity prevention/physical activity/nutrition (43.0%) (*P* = .02). A higher percentage of participants in tobacco control (63.3%) chose “improved health outcomes” in their top 3 than those working in diabetes or cardiovascular health (40.2%) (*P* = .002).

Rankings of perceived benefits did not differ by position (all *P* > .10, data not shown). Rankings for “consistency of messages” were higher among participants with more years in the agency (*P* = .04), and lower for “reduced duplication of program efforts” and “improved health department structure” (*P* = .04 and *P* = .02, respectively). Rankings were similar for state-level characteristics (*P* > .20), except a higher percentage of participants from early coordinated chronic disease prevention (CCD) planning states ranked “innovative approaches identified” in their top 3 than did those from other states (*P* = .04).

### Perceived challenges of coordinated chronic disease prevention

“Funding restrictions” (eg, funder requires specific performance measures, categorical funding) and “competing priorities” were the 2 most commonly and highest-ranked items for perceived challenges (Figure 2). Disease-specific partners include national and regional nonprofit organizations that focus on a specific set of conditions such as diabetes, cancer, or heart health.

For the most commonly ranked challenges, Table 2 compares the percentages of participants that chose the challenge as 1 of their top 3 items by participant program area. Participants did not differ by program area on the 2 highest ranked challenges, “funding restrictions” and “competing priorities.” Rankings for “lack of communication across programs” and “funding might be reduced” did differ by program area (*P* = .02 and *P *< .001, respectively). Table 3 provides illustrative quotes from survey participants for commonly ranked perceived challenges.

**Table 2:** Participants’ Perceived Challenges of Coordinated Chronic Disease Prevention, by Program Area, United States, 2013

Challenge	All (N = 865)^a^	Tobacco (n = 109)	Cancer (n = 93)	Diabetes or CVD (n = 92)	OPAN (n = 149)	Generalists^b^ (n = 129)	All Else (n = 293)	*P* Value^c^

	%							
Funding restrictions	49.2	46.8	44.1	48.9	53.0	54.3	47.8	.60
Competing priorities	53.9	57.8	55.9	50.0	56.4	53.5	51.9	.82
Lack of communication across programs	37.8	40.4	48.4	27.2	43.0	31.8	36.9	.02
Funding might be reduced	31.9	21.1	20.4	45.6	36.2	31.0	33.4	<.001
Agency not structured for CCD	26.0	31.1	23.7	25.0	25.5	22.5	27.0	.73
Loss of disease-specific partner support	23.9	15.6	25.8	25.0	22.8	29.5	24.2	.25

Abbreviations: CVD, cardiovascular disease prevention, cardiovascular health program; OPAN, obesity prevention, physical activity, and/or nutrition programs other than the Special Supplemental Nutrition Program for Women, Infants, and Children; CCD, coordinated chronic disease prevention.

a Percentage of participants that selected the response option as 1 of their top 3 challenges.

b Participants that checked 6 or more program areas.

c Determined using χ^2^ test.

**Table 3:** Illustrative Quotes About Participants’ Perceived Challenges to Coordinated Chronic Disease Prevention, United States, 2013

Challenge	Quote
Competing priorities	Lack of staff time to take on additional projects Achieving consensus among program areas Competition among diseases/conditions
Funding restrictions	Current funding goals, activities, do not provide funding or time to address core risk factors
Lack of communication across programs	Effective communication of priorities across various programs Integration requires superior communication skills and commitment
Funding might be reduced	Significant effort required to do well and there is less funding to do it Decreased staff capacity in light of funding cuts
Agency not structured for CCD	Lack of support from senior administrators Lack of vision regarding the benefit. . . . The existing culture does not promote such Agency is just too political especially in its decision making Lack of process ingenuity Integration is used to gain power and control over other programs
Loss of disease-specific partner support	Constituencies for primary prevention more diverse and may be harder to sustain support Loss of focus for specific policy interventions that require community interest and mobilization Community buy-in

Abbreviation: CCD, coordinated chronic disease prevention.

Rankings of perceived challenges were also assessed by position, years in public health, and state-level characteristics (data not shown). A higher percentage of managers and directors than staff ranked “disease-specific partners may not be as supportive” (27.6% vs 17.4%, *P* < .001) and “reduced public understanding” (6.3% vs 2.9%, *P* = .03) as perceived challenges. Fewer managers and directors ranked “lack of focus” as a challenge than did staff (7.8% vs 15.8%, *P* < .001). As years working in the state health department increased, rankings were higher for “disease-specific partners may not be as supportive” (*P* = .002) and lower for “lack of communication across programs” (*P* = .004) and “staff turnover” (*P* < .001). Challenges did not differ among states with early CCD planning versus other states (*P* > .20 except for “competing priorities” *P* = .07). Participants were more likely to rank “competing priorities” as a challenge in states with higher chronic disease prevalence (*P* = .03). Participants were more likely to rank “funding might be reduced” if they were from states with higher chronic disease prevalence (*P* = .01), lower spending on chronic disease (*P* = .004), and lower staff turnover (*P* = .002) and vacancy rates (*P* < .001).

“Other” challenges that participants typed in and ranked (n = 69, Figure 1) were categorized through consensus coding as

- Lack of leadership support (n = 16);
- Lack of staff time (n = 12);
- Lack of staff capacity (n = 7);
- Politicized funding or other decision making within the state health department (n = 3);
- Job insecurity (n = 2);
- Unclear vision (n = 2); and
- Turf issues (n = 2).

## Discussion

Information on perceived benefits and challenges from this national sample of state health department chronic disease practitioners may be useful for funding agencies, health departments, and training organizations for planning technical assistance to support transitions to coordinated chronic disease prevention. To realize the anticipated benefits and efficiencies and sustain coordination without designated funding, it will be important to overcome commonly experienced challenges.

Although literature to date is sparse, preliminary reports and success stories align with the anticipated benefits found in this sample. Several researchers found reduced duplication of efforts following coordination initiatives (8,15,21,22). For example, 91% of surveyed staff in Wisconsin’s Bureau of Community Health Promotion reported reduced duplication among coordinated programs in 2012 (15). There is also evidence of increased population reach through coordinated efforts in the Communities Putting Prevention to Work (CPPW) initiatives (23).

In the Colorado Department of Public Health and Environment, the Prevention Services Division established a policy coordination workgroup in 2011 (24). The workgroup maximizes resources by serving as a central contact point for access to policy and legal expertise, providing linkages with statisticians for evidence-based problem identification and evaluation improvements, identifying evidence-based policies on which to share resources, and coordinating outreach with community partners. The Colorado Department of Public Health and Environment also consolidated their epidemiology and evaluation programs into one branch and created a position dedicated to program coordination.

Frameworks of coordination across programs have facilitated the efficient use of resources and population reach in the United States, Canada, and Australia (8,11,21,25,26). Wisconsin staff members found that using a framework for coordination and a combined risk factor report was useful for thinking collaboratively and developing common messages across programs (15). In Alberta, Canada, collaborators developed a framework and common messages and posted them online to create a united purpose and further coordination (21). In the United States, Canada, and Australia, frameworks incorporate a socioecologic perspective with across-sector action and multilevel interventions.

Similar to our findings, overcoming lack of communication across programs is a commonly cited challenge and key element in coordination efforts in the United States, Canada, and Australia (11,21,22). Staff from Canada and Australia indicated the need and usefulness of in-service trainings on how to communicate across disciplines and across disease-specific coalitions (21,22). Wisconsin also used program evaluation data to guide communication improvements to increase program efficiency (15). Many states, including Connecticut, Massachusetts, and Missouri, have established cross-cutting workgroups in the state health department or a multiagency coalition to improve communication and address additional challenges to coordination (8,15,24,26–28). These workgroups can help overcome communication challenges, set common priorities, maximize resources, and address funding challenges. New York, Oregon, and others created communication guidelines to support shared visions and messaging. Oregon also established its Code of Collaboration on shared values and expectations of how people work together and incorporated it into new employee orientation. California hired a communications expert that conducted marketing research with state partners to support consistent messaging.

The creation of supportive administrative practices for new organizational structures, as has been done in North Carolina, New York, Oregon, and elsewhere, is one tool for overcoming the challenges of coordination (1,26,28,29). The Oregon Public Health Division restructured work teams and reclassified staff positions. Elements that Oregon staff reported as helping to overcome restructuring challenges were supportive leadership, nonmanagerial staff participating in coordination leadership, creation of a shared vision, in-person communication, and taking the time to manage the change process. Staff in Oregon perceived short-term coordination outcomes of increased efficiency both in the state health department and with partners (26). North Carolina found that a conducive agency infrastructure and centralized management capacity were important as were clear, consistent messages from the start (29). In Pennsylvania, the advisory committee for the Preventive Health and Health Services Block Grant was expanded to a Public Health Council (L. Best, written communication, December 2013). The Council provides guidance and recommendations for the coordinated chronic disease plan as well as a coordinated approach to implementation of the 4 domains and evaluation. Members are drawn from every categorical program and pertinent statewide advisory groups.

Coordination efforts have been implemented in other initiatives, such as CPPW. Recent examples at the federal level include CPPW (23), Community Transformation Grants, Healthy Communities Program, and Racial and Ethnic Approaches to Community Health. In CPPW, communities designed leadership teams in the beginning for policy, systems, and environmental interventions in tobacco, physical activity, and nutrition. The teams were “integral to long-term success and implementation of the Community Action Plan” (23). The Robert Wood Johnson Foundation has also promoted coordinated community approaches through programs such as Healthy Kids, Healthy Communities and Leadership for Healthy Communities. A recent review of Healthy Kids, Healthy Communities strategies indicated that the community partnerships were addressing CDC-recommended interventions at all socioecologic model levels (30).

Our study has limitations. First, the findings are time-sensitive. Responses about perceived benefits and challenges could change because of influences such as budget cuts or changes in competing priorities or following additional experience with coordinated approaches. Reliability testing showed that the benefit responses did indeed change in just 2 to 4 weeks; only half (53.2%) of respondents ranked at least 2 of the same 3 benefit response options when taking the survey 2 to 4 weeks later. Survey data collection occurred while state health department staffs were applying for combined funding, which may have affected responses. Data collection was completed in mid-May 2013 before funding levels for 2013–2014 were announced. Additionally, the study provides no information on which approaches to coordination are effective.

Findings from this study and lessons learned from states and other countries can be used by funders, health departments, and nongovernmental organizations for planning, training, and technical assistance. As funding for the coordinated effort across all chronic diseases ends in early 2014, we recommend that training and technical assistance specifically address the following areas: 1) enhancement of organizational support for coordinated approaches across program areas, 2) communication across programs, 3) lessons learned, 4) challenges in restructuring health departments for coordination and maximization of resources, and 5) sustainment of the framework of coordination. Other important training topics include benefits for organizational partners and evidence-based identification of common risk factors (10). CDC regional technical coordinators work across chronic disease grants and serve as liaisons between chronic disease directors in state health departments and grant program officers. This effort may increase consistent messaging across CDC programs. Through this regional approach, the sharing of lessons learned across states and peer-to-peer networking may help overcome common challenges. Technical assistance from CDC and CDC-contracted organizations can help ensure common frameworks and management plans and address communication.

To ensure states learn from one another and facilitate institutionalization of coordination, funders should play a leadership role. Funders should reflect coordination infrastructures in their proceedings and through the development of performance measures that focus on common risk factors. Funders can also ensure that requests for proposals from different units within the same agency are congruent with each other. Funders can provide or pay for technical assistance to ensure common frameworks, management plans, and messaging across programs within a state. Technical assistance on how to sustain coordination through multiple funding streams is especially pertinent to address funding challenges in a tight economy.

Ongoing surveillance, monitoring, and evaluation of the coordinated approaches will be important moving forward to ensure that anticipated benefits are realized and that lessons across states can be shared. Mixed methods that involve quantitative surveys, case studies, in-depth interviews, and tracking of risk factor and health outcome data will provide the needed information to guide future coordination efforts.
